# Does Frailty Modify the Associations of Late-Life Vascular Risk Factors With Incident Dementia?

**DOI:** 10.1093/geroni/igaf122.3918

**Published:** 2025-12-31

**Authors:** Jason Smith, A Richey Sharrett, Priya Palta, B Gwen Windham, Pamela Lutsey, Alden Gross, Jennifer Deal

**Affiliations:** UNC School of Medicine, Chapel Hill, North Carolina, United States; Johns Hopkins University, Baltimore, Maryland, United States; UNC School of Medicine, Chapel Hill, North Carolina, United States; University of Mississippi Medical Center - The MIND Center, Jackson, Mississippi, United States; University of Minnesota, Minneapolis, Minnesota, United States; Johns Hopkins Bloomberg School of Public Health, Baltimore, Maryland, United States; Johns Hopkins Bloomberg School of Public Health, Baltimore, Maryland, United States

## Abstract

The contribution of late-life vascular risk factors—hypertension in particular—to dementia remains controversial. Given the association of frailty with lower blood pressure (BP) specifically, we hypothesized that hypertension, but not diabetes or smoking, is more strongly associated with dementia risk in robust than in pre-frail/frail older adults. We used Cox proportional hazards models, adjusted for demographic and clinical/lifestyle characteristics, with a multiplicative interaction term between vascular risk factors and physical frailty (robust versus pre-frail/frail) to estimate cause-specific hazard ratios (HRs) of 11-year incident dementia associated with elevated BP, hypertension, diabetes and current smoking in community-living White and Black participants aged 67-89 years without dementia in the Atherosclerosis Risk in Communities Neurocognitive Study. We then stratified HRs of dementia by frailty. Overall, there were 377 (15.8%) dementia cases in robust participants (n = 2383; mean age, 74.2 years; 55.4% female) and 812 (30.0%) in pre-frail/frail participants (n = 2710; mean age 76.4 years; 61.3% female). There was an interaction between BP and frailty on dementia risk (P=.04). For robust participants, HRs were 1.06 (95% CI: 0.63-1.77) for elevated BP and 1.34 (95% CI: 0.92-1.94) for hypertension relative to normal BP. For pre-frail/frail participants, HRs were 0.59 (95% CI: 0.40-0.86) for elevated BP and 0.83 (95% CI: 0.67-1.04) for hypertension relative to normal BP. Diabetes and smoking were associated with increased dementia risk in both robust and pre-frail/frail individuals. Our findings suggest frailty status could be useful for clinical management of BP in older adults to support brain health.

